# Enteritis at Southwark Infirmary

**Published:** 1914-07-18

**Authors:** 


### Enteritis at Southwark Infirmary.

Dr. Bruce, the medical superintendent of the Southwark Infirmary, at the last meeting of the Southwark Board of Guardians reported that five cases of enteritis had occurred amongst the children in the infirmary, and that one was in a serious condition. We are afraid that this is but the prelude to a recurrence of the sad experience of twelve months ago, when there were over fifty cases of enteritis amongst the children, and when nine died. For the same conditions exist now as did then. Despite the opinion of the medical officer of the Local Government Board, and their suggestion that the Camberwell Borough Council should discontinue the use of the dust siding in close proximity to the infirmary, which, it was stated, was the direct cause of the outbreak, nothing has been done to remedy a condition of affairs which is detrimental, to say the very least, to public health. It would have been far more satisfactory if the Southwark Board of Guardians had grappled with the nuisance themselves, and not called in the aid of the Local Government Board, for they would long ere this have taken action in the interests of the community and have put a stop to such a disgraceful state of affairs; but, having called in the assistance of the Whitehall authorities, they are compelled to await their decision. It was thought that the intervention of the Local Government Board would have brought about a speedy termination of the nuisance, but it would appear that the very opposite has been the result, for the Local Government Board are still writing to the Camberwell Borough Council on the matter.

At the last meeting of the Southwark Guardians they had before them a copy of another letter which the Local Government Board had addressed to the Camberwell Borough Council. It stated that Dr. Hutchinson, one of their medical officers, had visited the siding near the infirmary. On the day he saw the siding there was no wind, and he could detect no smell in the grounds of the infirmary. The smell in the immediate vicinity of the siding was, however, very offensive, and there was a con siderable amount of house refuse and horse manure littered about, forming a continuous line of rubbish on both sides of the trucks. He found that the trucks were now half-screened during the process of filling, and are sheeted over when filled. These steps would tend to mitigate any nuisance, but he had no doubt that there was a nuisance at the time of his visit in the immediate neighbourhood of the refuse, which, with the wind in the right direction, might have been carried into the infirmary grounds. The letter stated that it would appear to the board desirable that the Council should make arrangements for the disposal of their refuse which would not involve the use of this siding. Until this could be done, the greatest care should be taken to obviate as far as possible the spilling of refuse in loading, and to collect and dispose of any refuse that is spilled. The Guardians decided to ask the Council to consider the matter before they adjourned for the summer recess, which lasts two months, but it is extremely unlikely that the Council will take any action. In the meantime, the lives of the children in the infirmary are to be endangered whilst all this letter writing, with its suggestions, goes on. Months of delay have taken place, and the last letter of the Local Government Board is only due to the intervention of Mr. E. A. Strauss, M.P., one of the members for Southwark, who threatened to raise the question in the House of Commons. It would appear that there is only one course open for the Southwark Board of Guardians, and that is to have the assistance of the law courts to get abated the nuisance which two medical officers of the Local Government Board have condemned.

